# Case Report: A case of successful treatment of STEMI following intra-coronary thrombolysis and re-transplantation in a heart transplant recipient

**DOI:** 10.3389/fcvm.2026.1835932

**Published:** 2026-07-27

**Authors:** Seok Hyun Kim, Soo Yong Lee, Min Ku Chon, Kook Jin Chun

**Affiliations:** Division of Cardiology, Department of Internal Medicine, Research Institute for Convergence of Biomedical Science and Technology, Pusan National University School of Medicine, Pusan National University Yangsan Hospital, Yangsan, Republic of Korea

**Keywords:** heart transplantation, intracoronary fibrinolysis, marinade technique, percutaneous coronary intervention, ST elevation myocardial infarction

## Abstract

**Background:**

The role of the Intracoronary fibrinolysis with distal occlusion (the Marinade technique) in managing refractory no-reflow during ST-segment elevation myocardial infarction in heart transplant recipients remains unknown.

**Case presentation:**

We report a case of a 38-year-old heart transplant recipient who presented with anterior ST-segment elevation myocardial infarction caused by total occlusion of the proximal left anterior descending artery. Despite repeated balloon dilations and thrombus aspiration, severe refractory no-reflow persisted and was complicated by ventricular fibrillation cardiac arrest requiring venoarterial-extracorporeal membrane oxygenation (VA-ECMO) support. Rescue intracoronary fibrinolysis using the Marinade technique—consisting of distal balloon occlusion and localized alteplase delivery with a prolonged dwell time—was repeatedly performed, resulting in partial restoration of coronary flow. Additional selective distal intracoronary alteplase administration via aspiration catheter successfully re-established distal coronary perfusion and thereby allowed completion of percutaneous coronary intervention (PCI). A total intracoronary alteplase dose of 25 mg was administered without major bleeding complications, despite concurrent VA-ECMO support. Although ST-segment resolution was limited, chest pain improved and hemodynamic stabilization was achieved, enabling successful redo heart transplantation.

**Conclusion:**

This case highlights the potential utility of intracoronary fibrinolysis using the Marinade technique as a bailout strategy for refractory no-reflow during PCI, even in high-risk settings such as heart transplant recipients with advanced microvascular dysfunction.

## Introduction

Cardiac allograft vasculopathy (CAV) in heart transplant recipients has a pathophysiology distinct from that of conventional atherosclerosis. Intracoronary fibrinolysis with distal occlusion (the Marinade technique) has recently been introduced as an effective strategy for treating the no-reflow phenomenon in patients with a heavy thrombus burden. Here, we report a case of a heart transplant recipient with ST-segment elevation myocardial infarction (STEMI) and cardiogenic shock requiring venoarterial extracorporeal membrane oxygenation (VA-ECMO), in whom coronary flow was restored using intracoronary fibrinolysis with distal occlusion.

## Case presentation

**Figure 1:**
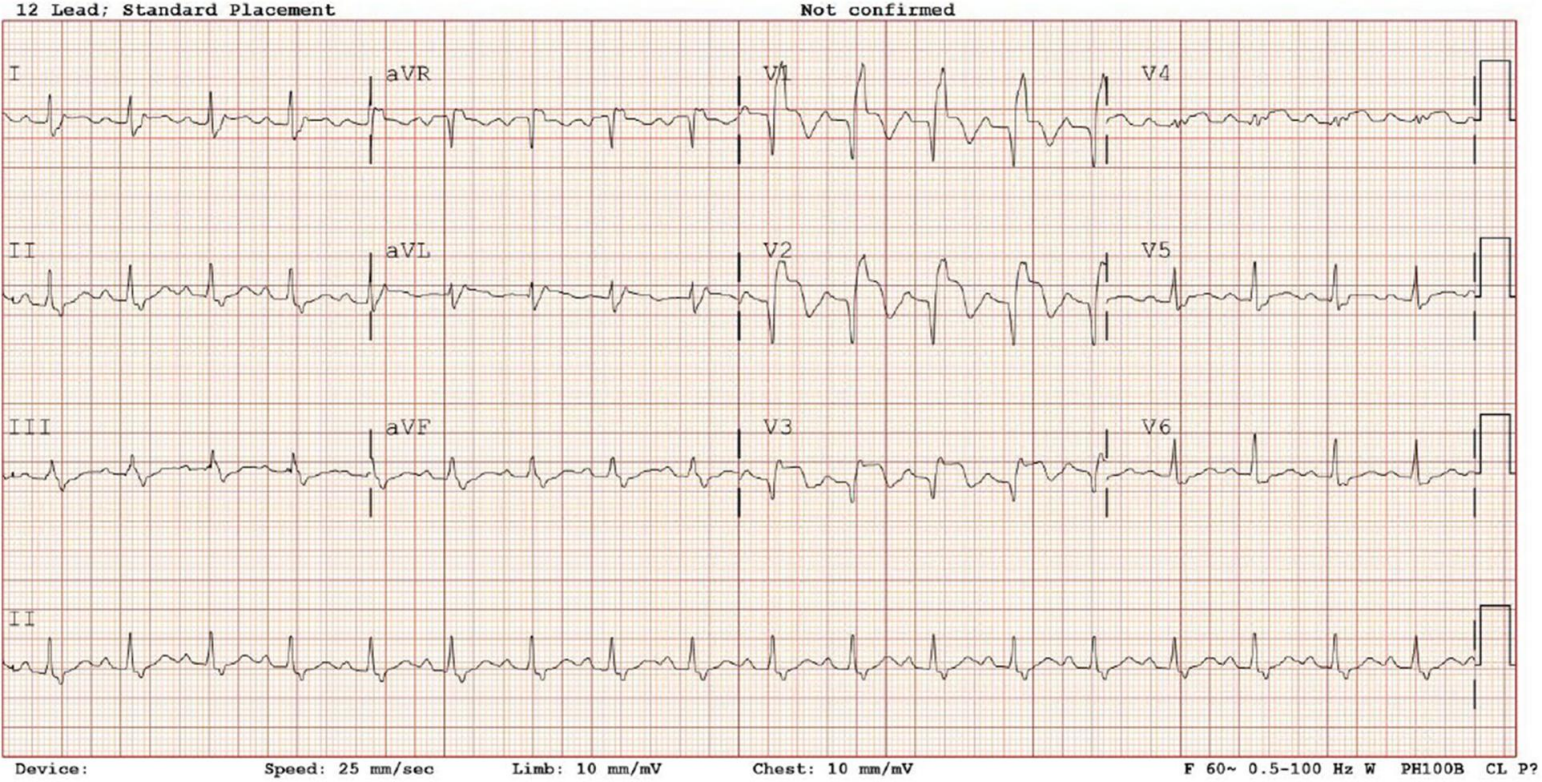
Initial electrocardiogram demonstrating ST-segment elevation in anterior leads.

A 38-year-old man presented to the emergency department with progressively worsening chest pain over three days. Initial vital signs were as follows: blood pressure 90/60 mmHg, heart rate 105 beats/min, respiratory rate 24 breaths/min, body temperature 36.6 °C, and oxygen saturation 98% on room air. The electrocardiogram demonstrated ST-segment elevation in the anterior leads (Figure 1).

The patient had undergone orthotopic heart transplantation eight years earlier for dilated cardiomyopathy, receiving a donor heart from an 18-year-old male with blood type B + . Routine surveillance coronary angiography performed 11 months prior revealed mild CAV. Due to financial constraints, intracoronary imaging was not performed at that time, and redo heart transplantation was planned if the disease progressed. He had been receiving immunosuppressive therapy, including everolimus and tacrolimus. He was on rosuvastatin/ezetimibe and the most recent low-density lipoprotein cholesterol level, measured one month before this visit, was 80 mg/dL.

**Figure 2:**
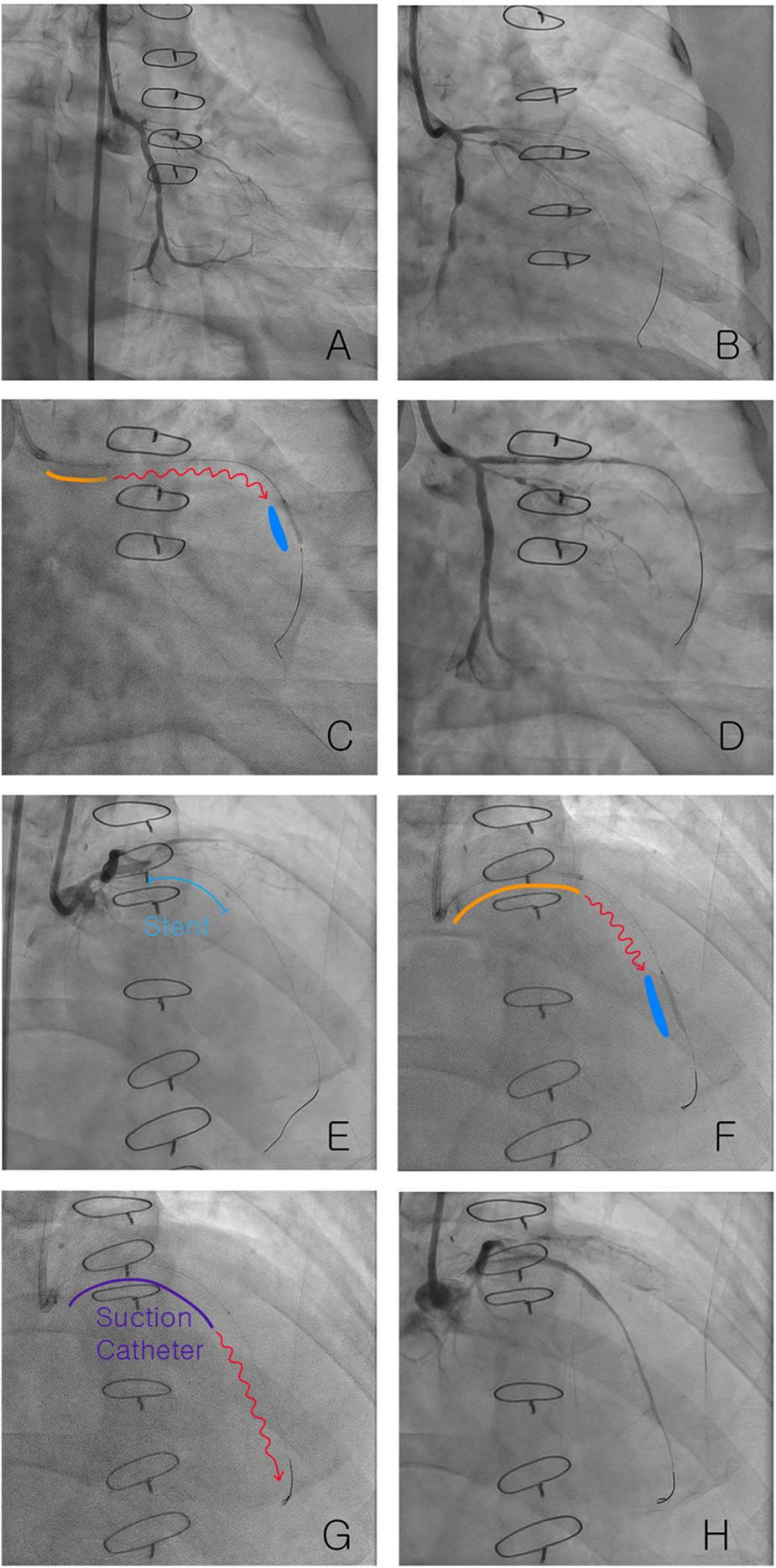
Percutaneous coronary intervention. **(A–D)** is from caudal view, while **(E–H)** is from cranial view. **(A)** Initial angiography showing total occlusion of the proximal LAD. **(B)** Angiography after multiple balloon predilations and thrombus aspiration, showing no improvement; immediately after intracoronary nicorandil (2 mg) injection, severe chest pain occurred, followed by ventricular fibrillation requiring defibrillation and initiation of VA-ECMO. **(C)** First Marinade technique session using a GuideLiner extension catheter positioned at the onset of no-reflow, distal occlusion with a 2.5-mm balloon, and intracoronary alteplase (5 mg) with a 5-minute dwell time. **(D)** Improved angiographic flow after two additional Marinade technique sessions using alteplase (5 mg each). **(E)** Angiography after intravascular ultrasound-guided deployment of a 2.75 × 33 mm drug-eluting stent, demonstrating recurrent no-reflow. **(F)** Additional Marinade technique sessions with alteplase (5 mg and 3 mg). **(G)** Persistent distal no-reflow treated with selective intracoronary alteplase (2 mg) via thrombus aspiration catheter. **(H)** Final angiography showing sluggish but adequate distal coronary flow (TIMI 2–3).

The patient was immediately loaded with aspirin and ticagrelor, followed by emergent coronary angiography, which revealed total occlusion of the proximal left anterior descending (LAD) artery. (Figure 2) Despite multiple balloon dilations and repeated thrombus aspiration, there was no improvement in coronary flow. Persistent no-reflow was observed, accompanied by gradual hypotension. At that point, ultrasound-guided puncture was performed to secure 5-Fr femoral arterial and venous access in preparation for mechanical circulatory support if needed. Immediately after intracoronary injection of nicorandil via thrombus aspiration catheter, the patient developed abrupt worsening of chest pain followed by ventricular fibrillation cardiac arrest. Manual chest compressions and defibrillation with 200 J were performed, and VA-ECMO was initiated. The patient achieved return of spontaneous circulation, with blood pressure of 72/57 mmHg and recovered consciousness.

However, distal coronary flow remained absent, and severe chest pain persisted. Based on prior successful experiences in managing refractory no-reflow, the operator elected to perform intracoronary fibrinolysis rather than administering additional intracoronary vasodilators. To restore coronary perfusion, intracoronary alteplase (5 mg) was administered through a 5.5 Fr GuideLiner catheter (Teleflex, Wayne, PA, USA), with distal occlusion achieved using a 2.5 mm balloon and a dwell time of 5 min (Marinade technique). Follow-up coronary angiography demonstrated restoration of distal flow for the first time. After intravascular ultrasound assessment, two more sessions of Marinade technique were required during the lesion preparation. Then, a drug-eluting stent (Xience Skypoint 2.75 × 33 mm, Abbott, Santa Clara, CA, USA) was deployed in the proximal LAD.

Subsequently, no-reflow recurred. Intravascular ultrasound showed no edge dissections. Additional Marinade technique sessions were performed with intracoronary alteplase (5 mg followed by 3 mg) at five-minute intervals. Flow was restored up to the stented segment; however, distal flow remained compromised. Therefore, alteplase (2 mg) was infused into the distal LAD through a thrombus aspiration catheter. This resulted in sluggish but definite restoration of distal flow, and the procedure was concluded (Supplementary Videos S1, S2).

**Figure 3:**
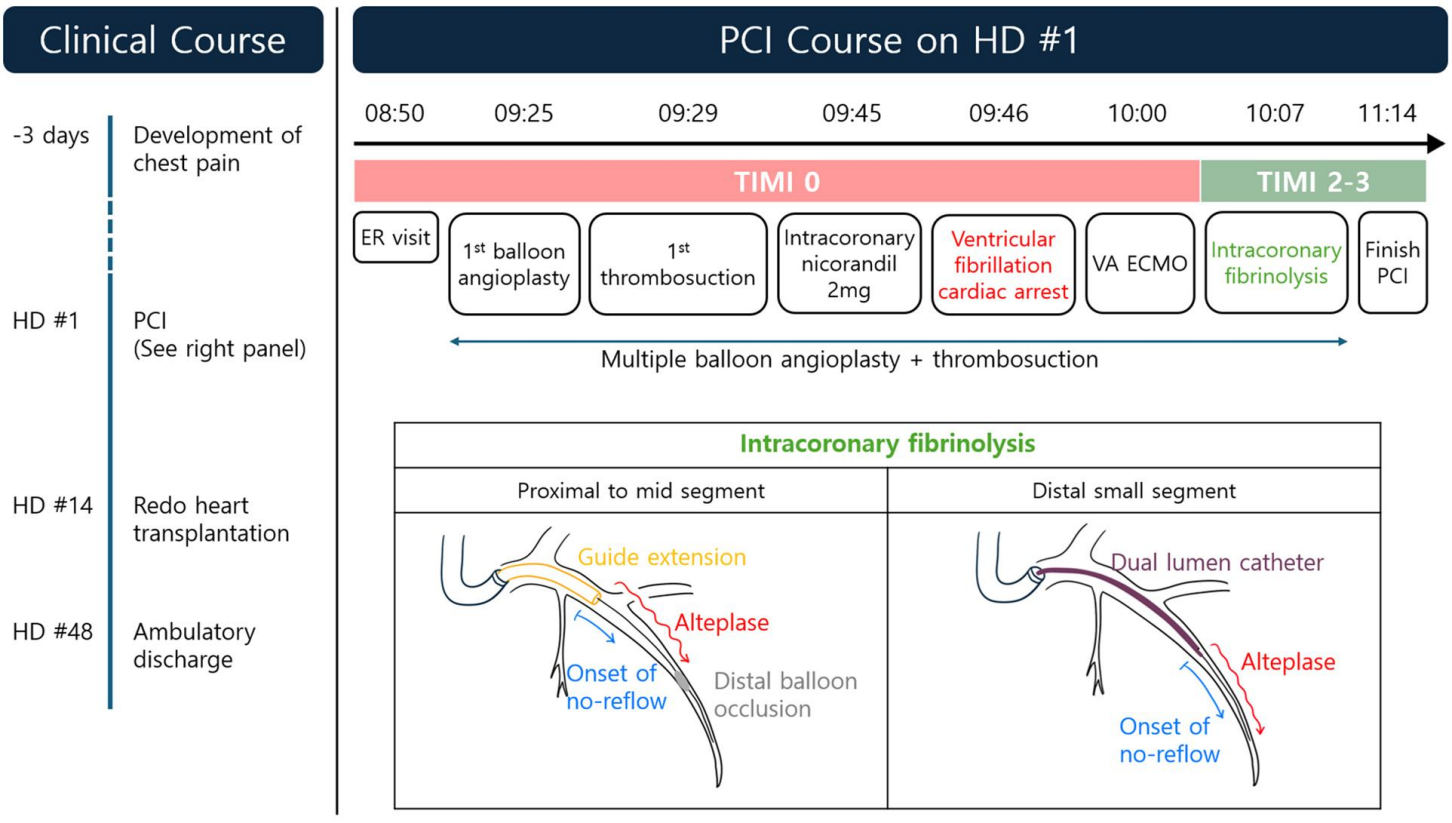
Clinical timeline and PCI course. The left panel summarizes the patient's overall clinical course from symptom onset to hospital discharge. The right panel illustrates the PCI course on hospital day 1, including the sequence of interventions, changes in TIMI flow grade, occurrence of ventricular fibrillation cardiac arrest, initiation of VA-ECMO, and intracoronary fibrinolysis for refractory no-reflow. ECMO, extracorporeal membrane oxygenation; ER, emergency room; HD, hospital day; PCI, percutaneous coronary intervention; TIMI, thrombolysis in myocardial infarction; VA, venoarterial.

Post-procedure, dual antiplatelet therapy and heparin infusion were maintained. Although ST-segment elevation showed limited resolution compared to the initial electrocardiogram, chest pain improved. The patient remained on VA-ECMO under intensive care. He was immediately listed for heart retransplantation (Status 1 according to the United Network for Organ Sharing and Status 0 according to the Korean Network for Organ Sharing) and successfully underwent redo heart transplantation on hospital day 14 with a donor heart from a 39-year-old male with blood type O + . After approximately one month of recovery, the patient was discharged ambulatory and continues outpatient follow-up (Timeline: Figure 3).

## Discussion

In heart transplant recipients, CAV affects not only the epicardial coronary arteries but also the microvasculature. Its pathophysiology is heterogeneous, ranging from conventional atherosclerosis to immune-mediated injury (1).

Coronary no-reflow is a well-known procedural factor associated with poor outcomes after percutaneous coronary intervention (PCI) (2). It is frequently observed in the presence of heavy thrombus burden, longer duration of preceding ischemia, endothelial swelling, microvascular spasm, among other mechanisms (3). Several strategies have been proposed to manage no-reflow, including intracoronary adenosine, epinephrine, verapamil, and glycoprotein IIb/IIIa inhibitors (4–6); intra-aortic balloon pump support may also have a potential role by improving microvascular obstruction after restoration of epicardial coronary patency (7).

Many patients with mild no-reflow or slow-flow phenomena can be successfully managed with intracoronary vasodilator administration. Consequently, most studies in this field have focused on evaluating pharmacologic agents or procedural strategies in large patient populations to determine whether they can improve TIMI flow, reduce infarct size, or prevent the occurrence of no-reflow. As a result, the available evidence primarily addresses the efficacy of individual therapies rather than the optimal management of refractory no-reflow once conventional treatments have failed (8–12). Reflecting the currently available evidence, a recently published review article proposed glycoprotein IIb/IIIa inhibitors as the principal second-line treatment option for no-reflow (13, 14). However, a small subset of patients develop severe refractory no-reflow, as observed in the present case, in whom conventional therapies, including glycoprotein IIb/IIIa inhibitors, may fail to restore distal coronary flow. Because such cases are uncommon and are typically reported only as case reports or small case series, adequately powered studies evaluating treatment escalation strategies have not been performed. Consequently, evidence-based recommendations for the management of refractory no-reflow remain limited. Particularly in patients with coronary ectasia, in whom heavy thrombus burden is common, PCI often fails to progress to definitive stenting during the index procedure, prompting consideration of staged stenting with systemic glycoprotein IIb/IIIa inhibitors. However, if the flow does not recover and thrombus hardens, rewiring of the distal vessel becomes extremely challenging, often resulting in failed revascularization and unfavorable clinical outcomes.

Leire Unzué et al. introduced an innovative intracoronary fibrinolysis strategy known as the “Marinade technique” (15). This technique involves advancing a guide extension catheter proximal to the thrombotic segment, occluding distal flow with a balloon, and administering a fibrinolytic agent with a dwell time of approximately five minutes. This approach allows marked reduction of thrombus burden and restoration of coronary flow.

Although intracoronary fibrinolysis has been previously investigated, prior studies primarily evaluated routine or preventive administration in acute coronary syndrome and failed to demonstrate statistically significant improvements in clinical outcomes (10, 16). Importantly, these studies did not focus on refractory no-reflow, nor did they employ the Marinade technique.

The key advantage of the Marinade technique lies in targeted drug delivery. By administering the fibrinolytic agent immediately proximal to the no-reflow segment with distal occlusion, both proximal backflow into the aorta and distal washout are minimized, allowing a higher local concentration of the drug to act directly on the thrombus (17).

Although concerns may arise regarding temporary distal coronary occlusion for approximately five minutes, our experience suggests that in patients with heavy thrombus burden and absent flow, temporary occlusion does not result in hemodynamic compromise. In contrast, attempting alternative techniques often consumes more time without meaningful benefit.

For no-reflow involving smaller distal coronary beds where balloon occlusion is not feasible, selective fibrinolytic spraying via thrombus aspiration catheters, dual-lumen microcatheters, or other dedicated devices may be an effective alternative.

Previous reports on the marinade technique have described intracoronary alteplase doses of up to 5 mg, whereas a randomized controlled trial administered doses as high as 20 mg (10). In this case, recurrent no-reflow at multiple procedural stages necessitated a cumulative intracoronary alteplase dose of 25 mg during a single intervention. Alteplase is a fibrin-selective fibrinolytic agent approved for acute myocardial infarction, pulmonary thromboembolism, ischemic stroke, and catheter thrombosis. It has a short half-life of approximately 3.5 min, resulting in rapid systemic clearance (18). Consequently, if no-reflow persists after an initial Marinade procedure or recurs following stent implantation, additional Marinade treatments can be performed immediately while directly assessing distal flow restoration. This feature represents a potential practical advantage over glycoprotein IIb/IIIa inhibitors, whose therapeutic effects are prolonged and cannot be readily reassessed during the same procedure. However, it may induce thrombin activation, raising concern for rebound thrombosis after washout. Therefore, we routinely maintain therapeutic heparinization according to acute myocardial infarction protocols for at least 24 h following use of this technique.

Another noteworthy aspect of this case is the safe administration of intracoronary fibrinolytics during VA-ECMO support. Securing femoral arterial and venous access under ultrasound guidance prior to hemodynamic collapse minimized vascular complications during ECMO initiation. Despite the relatively high cumulative dose of intracoronary fibrinolytics, no major bleeding complications, including access-site bleeding, were observed.

Several factors likely contributed to persistent no-reflow, including late presentation to the hospital, pre-existing CAV, transplant-related microvascular dysfunction, and acute myocardial infarction–associated injury (1, 3). Although the Marinade technique alone did not ultimately cure this patient, in a life-threatening situation characterized by refractory no-reflow and cardiac arrest, this technique effectively mitigated microthrombus-related no-reflow, restored coronary flow, relieved chest pain, and enabled hemodynamic stabilization until redo heart transplantation could be performed. This case suggests that the Marinade technique may be a valuable adjunct during PCI in heart transplant recipients.

## Conclusion

In a heart transplant recipient undergoing PCI for STEMI complicated by refractory coronary no-reflow, intracoronary fibrinolysis using the Marinade technique and selective distal fibrinolytic delivery via microcatheters proved to be effective strategies for improving coronary flow.

## Data Availability

The original contributions presented in the study are included in the article/Supplementary Material, further inquiries can be directed to the corresponding author.
